# A case report of human intoxication due to a snakebite by the opisthoglyphous dipsadid *Thamnodynastes lanei* Bailey, Thomas & Silva-Jr, 2005

**DOI:** 10.1590/0037-8682-0194-2020

**Published:** 2020-11-13

**Authors:** Pedro Henrique Salomão Ganança, Rafael de Fraga, Lourival Baía de Vasconcelos, Alfredo Pedroso dos Santos

**Affiliations:** 1 Universidade Federal do Oeste do Pará, Instituto de Ciências da Educação, Laboratório de Ecologia e Comportamento Animal, Santarém, PA, Brasil.; 2 Universidade Federal do Oeste do Pará, Instituto de Ciências e Tecnologia das Águas, Programa de Pós-Graduação em Biodiversidade, Santarém, PA, Brasil.; 3 Universidade Federal do Oeste do Pará, Programa de Pós-graduação em Recursos Naturais da Amazônia, Santarém, PA, Brasil.

**Keywords:** Amazonia, Public health, Tropical disease

## Abstract

We present a case of human intoxication due to a snakebite by the opisthoglyphous dipsadid *Thamnodynastes lanei.* A 26-year-old man was bitten on the right hand and was not medicated. Bleeding lasted a few seconds, while paresthesia, chills, and headache persisted for up to 10 hours. The pain disappeared after a week, and the edema, itching, and prickling persisted for another 3 days. Although this patient’s symptoms were typical of bites by South American opisthoglyphous snakes, they persisted longer than those of bites by some congeneric species. Our report adds a species to the list of medically relevant snakes.

## INTRODUCTION

Snakebite has been considered a priority neglected tropical disease by the World Health Organization, since the incidence and severity are high enough to generate a demand for specific public health policies^1^. Snakebites are also a matter of social interest since most patients are poor people who live and work in rural areas^1^. The first step towards effective public health policies is to determine the list of snakes that health professionals should be familiar with for identifying species and bite symptoms. However, this may not be a straightforward task when considering opisthoglyphous snakes because there is a debate about their classification in relation to public health, since symptoms caused by the bites of these snakes vary widely among species and patients^3^.

Opisthoglyphous snakes have traditionally been classified as non-venomous snakes and are considered to be harmless to humans^2^. However, some species of the Colubridae and Dipsadidae families may cause local (e.g., bleeding and swelling) and systemic symptoms (e.g., fever and sweating), and even death, although a few cases are anecdotal^2^. Although biochemical and epidemiological studies on South American pitvipers (e.g., *Crotalus* and *Bothrops*) are relatively common, the potential toxicity of Colubridae and Dipsadidae families is poorly known^3^ ^,^ ^4^. Therefore, it can be assumed that snakebite incidence and severity of the opisthoglyphous species have been underestimated and cases studies are important for refining public health policies^3^ ^,^ ^5^.

Among the South American opisthoglyphous snakes, the genus *Thamnodynastes* is relevant to public health. The bites of species within this genus may cause bleeding, local pain, and edema, although symptoms tend to disappear within 3-8 days^3^ ^,^ ^6^ ^,^ ^7^. Several of the *Thamnodynastes* species share general characteristics such as beige color, slender body, long tail, short head, large eyes, and elliptical pupils^8^. Therefore, differentiating species may not be a straightforward task, especially for health professionals unfamiliar with taxonomy. This is particularly critical for snakebite epidemiology because symptoms may vary among species, patients, or even among regions within a species geographic range^9^. Even though symptoms are usually mild, medical reports are likely to be biased by species misidentification^9^ ^,^ ^10^ *.* It is not uncommon for healthcare professionals to misidentify Dipsadidae and Colubridae snakes as pitvipers^3^. Therefore, it is possible that there are unreported cases of *Thamnodynastes* bites that have caused more severe symptoms than those reported in the literature. Additionally, a combination of relatively severe symptoms of bites by opisthoglyphous snakes such as *Thamnodynastes* and misidentification of Dipsadidae and Colubridae species as pitvipers may lead to unnecessary antivenom-based treatment.

Although snakebite case studies involving *Thamnodynastes* have been published^3^ ^,^ ^6^ ^,^ ^7^, some species within this genus are still completely unknown in relation to the potential toxicity. Here, we report the symptoms of a bite by *Thamnodynastes lanei*, which occupies the grassy vegetation often associated to aquatic ecosystems in Argentina, Bolivia, Paraguay, and Brazilian Amazonia, Pantanal, and western Cerrado^8^. We focused on providing a chronological report of symptoms, with the main objective of informing health professionals and the general population about the toxic potential of this snake.

## CASE REPORT

**FIGURE 1:**
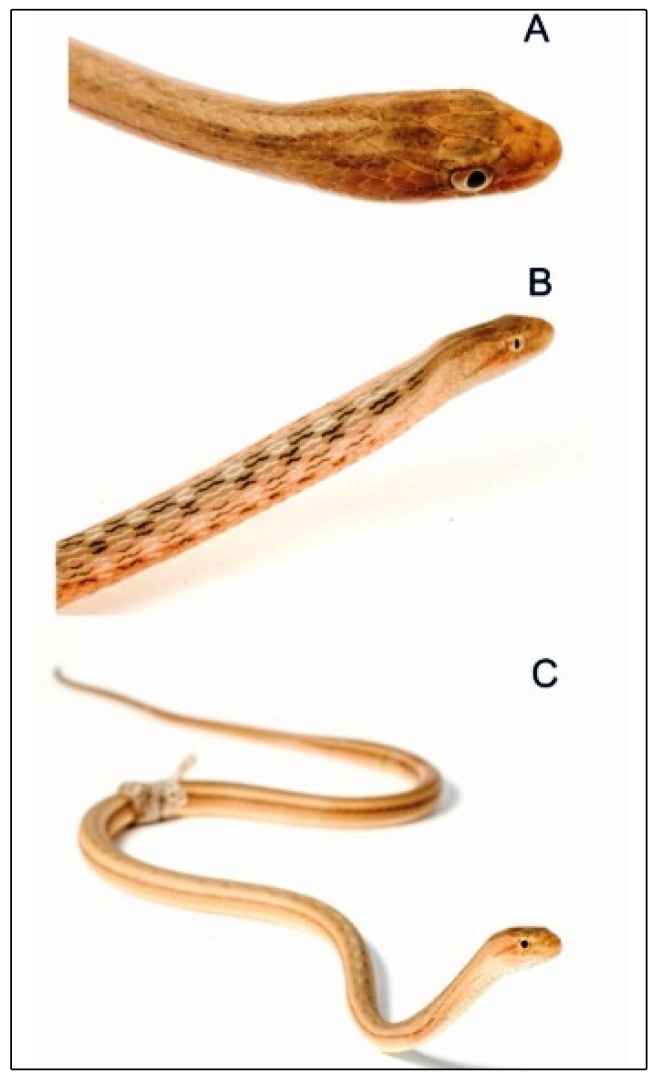
Unsexed adult *Thamnodynastes lanei* from a floodplain lake in western Pará, Brazil. **(A)** Details of the head, **(B)** flat-neck defensive posture, and **(C)** general view of body shape and color. Photos by Francesca Nicole Angiolani Larrea.

**FIGURE 2:**
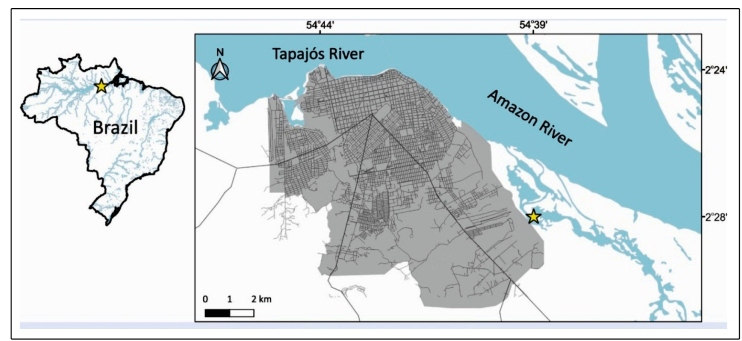
Santarém region (western Pará, Brazil). The yellow star denotes the location where the snakebite by *Thamnodynastes lanei* was reported.

A 26-year-old man (75 kg, 1.82 m) was bitten on the dorsal side of his right hand by an adult *T. lanei* (unsexed; snout-vent length, 380.7 mm; tail length, 130.9 mm; mass, 22 g; Figure 1). The snakebite occurred in a floodplain lake in the Municipality of Santarém, Pará, Brazil (2°30'42.02" S; 54°34'36.86" W; Figure 2), on April 18, 2019, at 9:46 pm. The patient is a herpetologist (senior author), who was bitten during data collection for a study on the ecology of reptile and amphibian assemblages occupying aquatic macrophyte banks. The contact of the rear fangs with the patient’s hand lasted about 40 seconds, which suggests aggressive behavior. We identified the species based on the diagnostic characters proposed in the original description of the species^8^. The specimen collected was deposited in the herpetological collection of the Universidade Federal do Oeste do Pará (UFOPA), Santarém, Brazil (voucher code UFOPA-H 2096). Our specimen collection protocols were authorized by IBAMA/ICMBio/SISBIO, process nº 24072-1, and the Ethics Committee of UFOPA (process Nº 1120180049).

**FIGURE 3:**
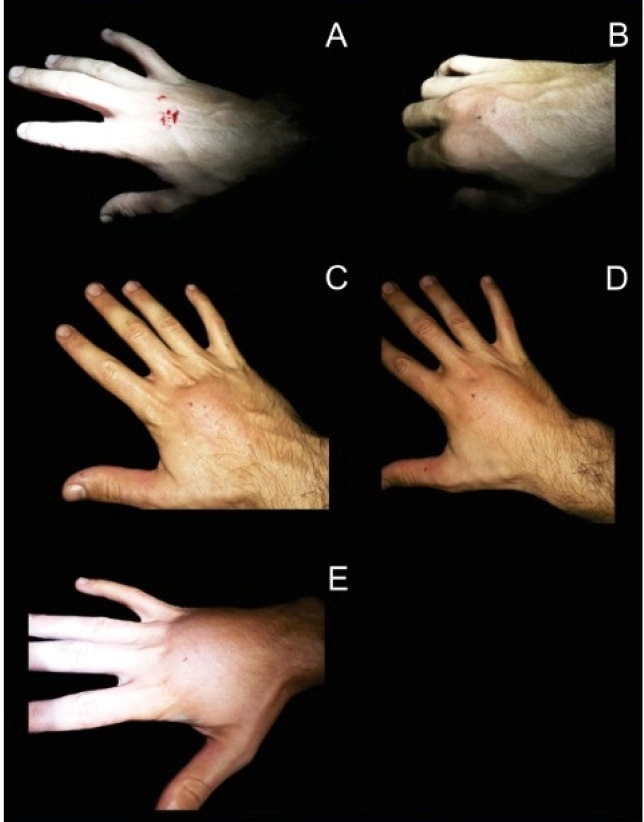
Chronological evolution of *Thamnodynastes lanei* snakebite symptoms. **(A)** 10 seconds, **(B)** 5 minutes, **(C)** 40 minutes, **(D)** 50 minutes, and **(E)** 2 hours and 30 minutes after the bite.

The first symptoms (0-10 seconds) were local swelling, mild pain, and bleeding (Figure 3). The next 7 minutes were marked by moderate pain, swelling, paresthesia, itching, prickling and burning sensations. Those symptoms became severe in the next 15 minutes and were followed by edema, headache, and chills. The edema spread to the entire dorsal side of the hand and the index finger in 50 minutes, and all fingers and wrist in 4 hours. At that point, the local pain decreased, and the headache and chills disappeared. After 10 hours since the bite, the edema reduced to intense pain, and paresthesia and prickling persisted only during muscle activity. The pain completely disappeared after a week, and the swelling, itching, and prickling persisted for another 3 days. We conclude that in the absence of medication and just washing the wound with soap and water, the symptoms of *T. lanei* bite can persist for up to 10 days.

## DISCUSSION

The symptoms that we report were qualitatively expected for intoxication by Dipsadidae snakebite^3^. However, the persistence time of the symptoms was more than three times longer than that in most reported cases of *Thamnodynastes* bite ^3^ ^,^ ^6^ ^,^ ^7^. In the literature, a case of single bite by a *Thamnodynastes* species has been characterized by symptoms persisting for 8 days, but most similar patients did not show symptoms for more than 3 days^3^ ^,^ ^6^ ^,^ ^7^. Additionally, the edema caused by *T. lanei* bite is visually larger than those caused by congeneric species^6^ ^,^ ^7^. These findings suggest interspecific variations in the biochemical structure of the *Thamnodynastes* venom, although immune responses are expected to vary among patients or geographic regions^9^. Therefore, the list of opisthoglyphous snakes that are relevant for public health (because they cause severe symptoms or are confused with pitvipers) should be constructed at species level. This highlights the relevance of timely cases studies, and the relevance of accurate taxonomic identification in reporting snakebites.

Although we found unique immune responses among reported cases of *Thamnodynastes* bites, we cannot quantify the effects on symptoms of unmeasured variables, such as the amount of injected venom and the patient's physical condition^11^. However, we argue that the severity and persistence of the symptoms reported here are high enough for *T. lanei* to be considered a medically relevant species. Even if the most severe symptoms are mild for most people, a combination of bleeding, pain, and edema could easily lead an inexperienced health professional to identify the case as a pitviper bite^3^. This would lead to waste of antivenom, which is a traditionally scarce product in rural South America, where it is most needed. Further, unnecessary antivenom-based treatment could put patients at risk of allergenic responses and anaphylactic shock^3^. Ultimately, the symptoms reported here should be part of a general guide for identifying bites by opisthoglyphous snakes, which would be used for both, monitoring the toxic potential of these snakes and for avoiding unnecessary antivenom-based treatment.

Snakebite is considered a priority neglected tropical disease by the World Health Organization (WHO) and a public health concern in South America^12^ *.* The epidemiology of snakebites is particularly underdeveloped in Amazonia, where the incidence is relatively high and underreported^11^, and large distances to urban centers can make the search for hospitals highly costly in terms of travel time and fuel costs. While part of the rural population tends to prefer traditional medicine to hospital admission^11^, many people may decide to travel unnecessarily to treat a bite from a *T. lanei* snake, as the symptoms can be confused with severe cases of pitviper envenomation. Therefore, this report is relevant from clinical and epidemiological points of view as well as educational. 
